# Genetic alteration and mutation profiling of circulating cell-free tumor DNA (cfDNA) for diagnosis and targeted therapy of gastrointestinal stromal tumors

**DOI:** 10.1186/s40880-016-0131-1

**Published:** 2016-07-21

**Authors:** Weixin Yan, Aiguo Zhang, Michael J. Powell

**Affiliations:** Robotics Research Institute, School of Mechanical Engineering, Shanghai Jiao Tong University, Shanghai, 200240 P.R. China; DiaCarta Inc., 2600 Hilltop Drive, Richmond, CA 94806 USA

**Keywords:** Gastrointestinal stromal tumors, Liquid biopsy, Mutations, Targeted therapy

## Abstract

Gastrointestinal stromal tumors (GISTs) have been recognized as a biologically distinctive type of tumor, different from smooth muscle and neural tumors of the gastrointestinal tract. The identification of genetic aberrations in proto-oncogenes that drive the growth of GISTs is critical for improving the efficacy of cancer therapy by matching targeted drugs to specific mutations. Research into the oncogenic mechanisms of GISTs has found that these tumors frequently contain activating gene mutations in either platelet-derived growth factor receptor A (*PDGFRA*) or a receptor tyrosine protein associated with a mast cell growth factor receptor encoded by the *KIT* gene. Mutant cancer subpopulations have the potential to disrupt durable patient responses to molecularly targeted therapy for GISTs, yet the prevalence and size of subpopulations remain largely unexplored. Detection of the cancer subpopulations that harbor low-frequency mutant alleles of target proto-oncogenes through the use of molecular genetic methods, such as polymerase chain reaction (PCR) target amplification technology, is hampered by the high abundance of wild-type alleles, which limit the sensitivity of detection of these minor mutant alleles. This is especially true in the case of mutant tumor DNA derived “driver” and “drug-resistant” alleles that are present in the circulating cell-free tumor DNA (cfDNA) in the peripheral blood circulation of GIST patients. So-called “liquid biopsy” allows for the dynamic monitoring of the patients’ tumor status during treatment using minimally invasive sampling. New methodologies, such as a technology that employs a xenonucleic acid (XNA) clamping probe to block the PCR amplification of wild-type templates, have allowed improved molecular detection of these low-frequency alleles both in tissue biopsy samples and in cfDNA. These new methodologies could be widely applied for minimally invasive molecular testing in the therapeutic management of GISTs.

## Background

Gastrointestinal stromal tumors (GISTs) are the most common mesenchymal tumors of the gastrointestinal tract, accounting for approximately 20% of all sarcomas [1, 2]. Sarcomas are a rare type of cancer that can occur in the bones, muscles, nerves, blood vessels, connective tissue, fat, and cartilage. The neoplastic GIST cell appears to arise from a common precursor cell, which gives rise to the interstitial cells of Cajal in the normal myenteric plexus [3]. GISTs are characterized by mutations in a receptor tyrosine protein (encoded by the *KIT* gene) associated with a mast cell growth factor receptor or in the gene encoding platelet-derived growth factor receptor A (*PDGFRA*). These genetic aberrations lead to constitutive activation of these growth factor receptors and concomitant abnormal cellular proliferation, which leads to the development of GIST.

Early detection of these genetic alterations is important for diagnosis and therapy and for monitoring the progression of GIST. This article provides an overview of the current status of targeted molecular therapies for GISTs and the current state-of-the-art of high-sensitivity molecular genetic tests that are able to detect low-frequency tumor-derived mutations in cancer patients employing minimally invasive sampling, i.e., “liquid biopsy”.

## Kinase mutations in GISTs

**Fig. 1:**
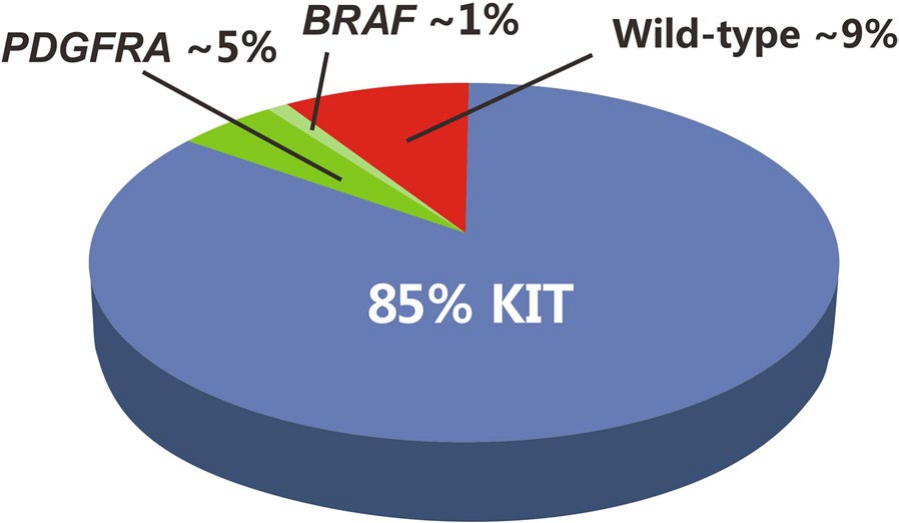
Molecular subsets of gastrointestinal stromal tumors (GISTs). Gene that encodes a receptor tyrosine protein associated with a mast cell growth factor receptor (*KIT*); platelet-derived growth factor receptor A (*PDGFRA*); gene that encodes an enzyme belonging to the RAF family of serine-threonine protein kinases participating in the RAS-RAF-ERK pathway (*BRAF*); wild-type (WT) normal gene sequence that does not contain any somatic mutations

The biology of GISTs has been widely investigated at the genomic level. Mutations in *KIT* or the receptor tyrosine kinase *PDGFRA* are the hallmarks of molecular diagnosis of GIST. *KIT* and *PDGFRA* are mutated in approximately 85% and 5%, respectively, of GISTs. Mutations are also rarely (<1%) found in the serine-threonine protein kinase *BRAF* (Fig. 1) [4].

**Fig. 2:**
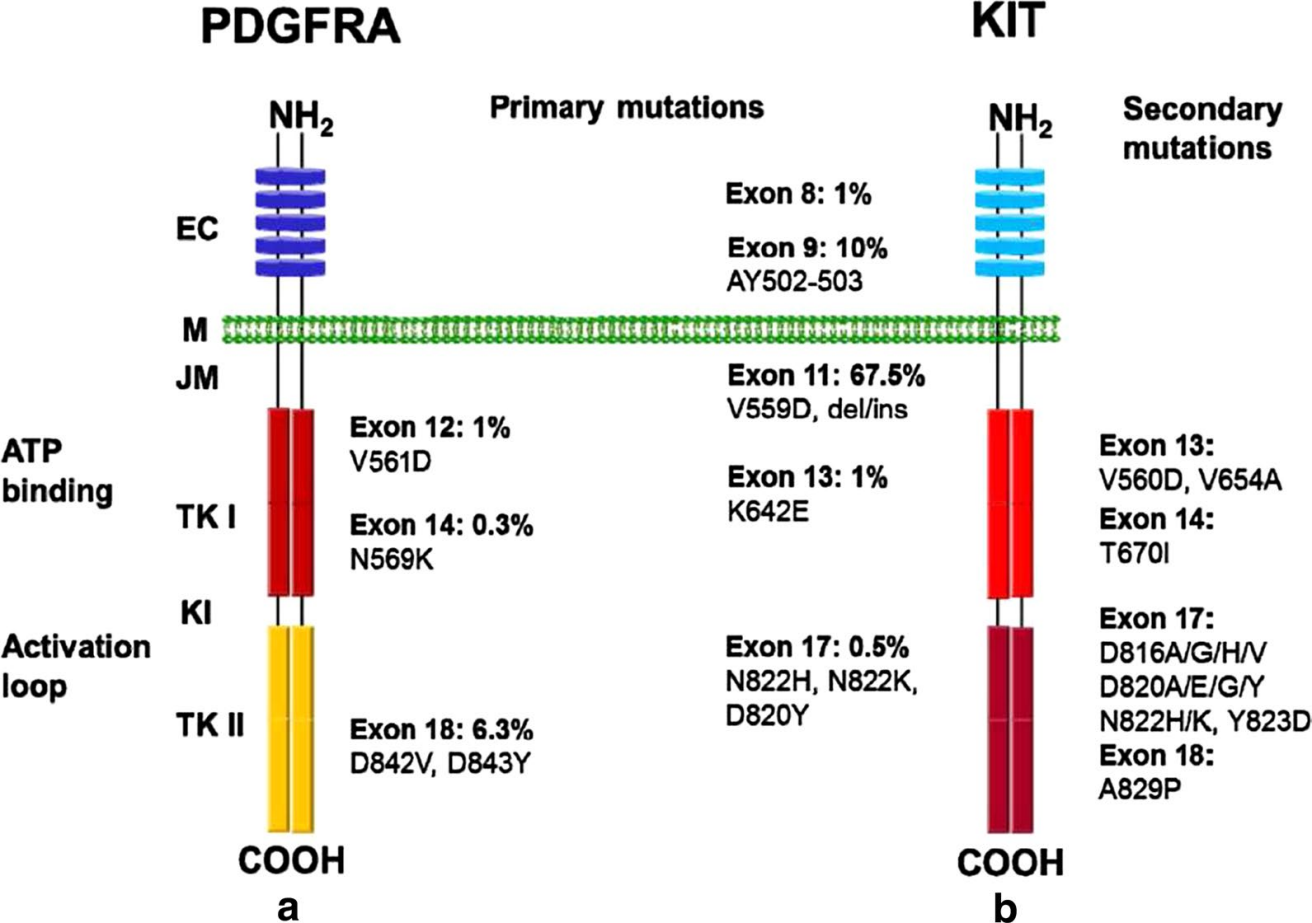
Schematic structures of *PDGFRA* and *KIT*. **a** Primary oncogenic mutation in *PDGFRA*. **b** Distribution of primary and secondary mutations in *KIT*. *EC* extracellular domain, *M* membrane, *JM* juxtamembrane domain, *TK I* tyrosine kinase domain I, *KI* kinase insert, *TK II* tyrosine kinase domain II

Approximately 80% of GISTs have an oncogenic mutation in the tyrosine kinase *KIT* [5–7]. Most of these mutations affect the juxtamembrane domain encoded by *KIT* exon 11, allowing spontaneous (ligand-independent) receptor dimerization and kinase activation. However, mutations also occur in exons 9, 13, and 17, and these mutations may support constitutive *KIT* signaling through other mechanisms. A subset (5%–7%) of GISTs have an activating mutation in the *KIT*-homologous tyrosine kinase *PDGFRA* [8, 9]. Many of these *PDGFRA*-mutant GISTs have an epithelioid morphology and express little or no KIT; however, such features are not unique to these tumors, and mutation status can be determined only through molecular analysis. Approximately 10%–15% of GISTs are negative for *KIT* and *PDGFRA* gene mutations; these tumors are often referred to as wild-type GISTs (Fig. 2) [10, 11].

Mutant and wild-type GISTs show marked differences in imatinib response. The response rates were approximately 80% in GIST patients with *KIT* exon 11 mutants, 40% in those with *KIT* exon 9 mutants, and 14% in wild-type GIST patients [12]. GIST patients with *PDGFRA* mutations showed mild sensitivity to imatinib (66%), with the exception of those with the exon 18 point mutation D842V, who were totally resistant to imatinib [12]. In addition to the importance of GIST mutational status in predicting imatinib sensitivity, as described above, the acquisition of secondary mutations in either *KIT* or *PDGFRA* represents the most frequent mechanism of imatinib resistance in GISTs. Advances in whole genome analysis technology has shown that GISTs with wild-type *KIT*/*PDGFRA* should be considered more appropriately as a heterogeneous family of distinct disease entities, with different biological and clinical features [13].

BRAF belongs to the RAF family of serine-threonine protein kinases. These kinases participate in the RAS-RAF-ERK pathway, which regulates cell cycle through activation of the mitogen-activated protein kinase (MAPK) pathway. *BRAF* mutations have been detected in GIST patients with wild-type *KIT*/*PDGFRA*. In a recent study, *BRAF* exon 15 V600E mutation was detected in 13% (9 of 70) of patients with wild-type *KIT*/*PDGFRA* GISTs [14]. In naïve GIST patients carrying activating mutations in *KIT* or *PDGFRA*, a concomitant activating mutation in *BRAF* gene (in approximately 2% of the patients) was found to be consistent with resistance [15]. In this study, in vitro experiments showed that imatinib was able to switch off the mutated activated receptor *KIT* but not the downstream signaling triggered by the *BRAF* mutation [15].

The most common primary mutations in *KIT* affect the juxtamembrane domain encoded by exon 11. Two-thirds of GISTs harbor *KIT* mutations in exon 11, which disrupt the normal juxtamembrane secondary structure that prevents the kinase activation loop from swinging into active conformation [16]. These mutations include in-frame deletions, insertions, and substitutions, as well as combinations of these. The deletions are associated with shorter progression-free and overall survival in comparison to other exon 11 mutations. In particular, deletions involving codon 557 and/or codon 558 are associated with malignant behavior [17].

Aside from exon 11 mutations, 7%–10% of GISTs have a *KIT* mutation in an extracellular domain that is encoded by exon 9. These mutations are thought to mimic the conformational change that the *KIT* receptor undergoes when stem cell factor (SCF) is bound. Importantly, the kinase domain in exon 9-mutant *KIT* is essentially the same as in wild-type *KIT* and has an effect on inhibitor sensitivity. Also important is that these mutations occur in tumors that arise in the intestines but are rarely seen in the stomach. *KIT* mutations in the activation loop (which is encoded by exon 17) of the kinase are uncommon and stabilize the active conformation. *KIT* mutations in the ATP-binding region encoded by exon 13 (such as K642E) are also uncommon. Secondary mutations are concentrated in two regions of the *KIT* kinase domain. One target is the ATP-binding pocket, encoded by exons 13 and 14, the part of the protein that directly interferes with drug binding. The other target is the activation loop, where mutations can stabilize *KIT* in the active conformation and thereby hinder drug interaction. By contrast, the secondary ATP-binding pocket mutations do not cause intrinsic kinase activation.

Crystal structure analysis of *KIT* has shown that in the absence of the ligand, the juxtamembrane domain folds back into the active site of the kinase. Disruption of the juxtamembrane domain by mutation is believed to lead to activation of the kinase by removal of this auto-inhibition. The prognostic significance of mutations in the *KIT* and *PDGFRA* genes has been examined in GISTs in the pre-imatinib era, and tumors with a *KIT* exon 11 mutation are associated with a worse outcome than tumors with other *KIT* or *PDGFRA* mutant isoforms or with no detectable mutation [18–22]. Conversely, *KIT* exon 11 mutations have been found in mitotically inactive GISTs of 1 cm or smaller, suggesting that oncogenic *KIT* activity contributes to early tumor growth [23, 24]. GISTs with a *KIT* exon 9 mutation arise predominantly in the small intestine and colon and appear to be clinically more aggressive than tumors with *KIT* exon 11 mutations [25, 26]. In contrast, tumors with *PDGFRA* mutations are less aggressive than those with *KIT* mutations [27, 28].

## Current targeted therapies for GISTs

The golden standard GIST therapy is the tyrosine kinase inhibitor (TKI) imatinib, which offers a good and stable response for approximately 18–36 months; however, development of resistance is very common. The detection of somatic mutations from cancer genome sequences is the key to understanding the genetic basis of disease progression, patient survival, therapy response, and toxicity. The DNA of the tumor serves as the primary source of information, which over the years has led to a more detailed characterization of the biological profile of GIST.

**Fig. 3:**
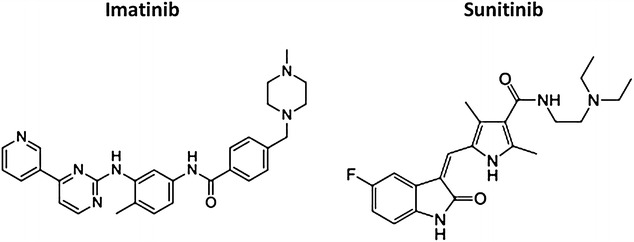
Chemical structures of the tyrosine kinase inhibitors (TKIs) imatinib [Gleevec, approved by the Food and Drug Administration (FDA) of the United States in 2002 for the treatment of advanced GIST patients and marketed by Novartis] and sunitinib (Sutent, approved by the FDA in 2006 for the treatment of patients with imatinib-resistant GIST and marketed by Pfizer)

For GISTs, the main objective of treatment is complete resection. As discussed above, TKIs such as imatinib or sunitinib (Fig. 3) are used for neoadjuvant, adjuvant, or palliative treatment. Sunitinib malate is an oxindole molecule designed to interact selectively with the intracellular ATP-binding sites of tyrosine kinase vascular endothelial growth factor receptors 1–3 (VEGFR1–3), platelet-derived growth factor receptors (PDGFRs), stem cell growth factor receptor (KIT), fms-related tyrosine kinase 3 (FLT3), and colony-stimulating factor 1 receptor (CSF1R).

Receptor inhibition has multiple effects on cellular processes, including tumor cell survival, endothelial cell growth and migration, vascular permeability, pericyte recruitment, and lymphangiogenesis. The final antitumor effects may be classified as follows: direct cytotoxic effects on tumor cells by induction of cell death, anti-angiogenic effects leading to growth delay and/or tumor regression by cytostatic inhibition of new blood-vessel formation, vascular disruption by inhibition of existing VEGF/VEGFR-dependent tumor blood vessels leading to central tumor cell necrosis, and cavitation that may or may not be associated with tumor regression. Responses to imatinib can be achieved; however, treatment is not curative unless complete resection is possible. In advanced GISTs, a partial remission can be attained in 50% of patients treated with imatinib, and there are single cases of complete responses. However, most patients receiving imatinib experience disease progression [29–34]. Patients receiving imatinib who progress only transiently respond to sunitinib [6, 35, 36].

For patients with GISTs, the response to therapy is evaluated by diagnostic imaging, which displays limited sensitivity and specificity. Computed tomography (CT) constitutes the golden standard of imaging for GIST [37, 38]. However, sensitivity of CT in detecting GISTs is insufficient. Currently, there is no biomarker available for detecting success or failure of therapy in GISTs. Activating mutations in *KIT* or *PDGFRA* can be found in at least 85% of all GISTs and constitute the transforming event in the pathogenesis of GIST tumors. In addition, mutated genomic DNA fragments are highly specific for the tumor. High sensitivity methods for the detection of these mutations are needed. Currently, there is limited clinical sensitivity of Sanger dideoxy sequencing; at least ~20% of the DNA derived from the tumors needs to be detectable mutants, meaning that GISTs harboring mutations below this threshold will go undetected.

## Mutation detection of circulating cell-free tumor DNA (cfDNA) for diagnosis and cfDNA-targeted therapy for cancer

Circulating cfDNA can be detected in healthy individuals but is increased in patients with tumors [39–43]. Proposed models for tumor DNA release into the blood include DNA release by tumor cells that undergo apoptosis or necrosis and extravasation of tumor cells into the blood, where cells undergo lysis.

It has been shown that the levels of long interspersed nucleotide elements 1 (LINE1) DNA in plasma samples associate with tumor progression in different cancer entities [44–47]; however, LINE1 sequences are not tumor-specific. It has also been shown that growth factor mutations can be recovered in cfDNA in serum samples of patients with non-small cell lung cancer [48], and detection of circulating mutant tumor suppressors adenomatous polyposis coli (*APC*) and *p53*, or *KRAS* DNA was shown to associate with progression-free survival in patients with colorectal cancer [49].

## cfDNA in GISTs

Understanding the genetic landscape of GISTs is important for selection of the most appropriate medical treatment. A sensitive, reliable biomarker for detection and quantitation of disease activity would be a highly valuable tool for the management of GIST. Traditionally, DNA extracted from formalin-fixed, paraffin-embedded specimens has been used for genetic analysis. However, a recent study showed that tumor-specific mutations in *KIT* or *PDGFRA* can be detected and quantified in circulating cfDNA in plasma samples from patients with GIST. This technique is known as “liquid biopsy” [50]. A target amplification methodology known as quantitative ligation polymerase chain reaction (PCR) was used, and the cfDNA levels were associated with the clinical course of the GIST lesions as measured by diagnostic imaging. Thus, tumor-specific cfDNA in plasma can be used as a highly specific biomarker in patients with GIST and can also be used to predict treatment responses and relapse, allowing for earlier changes in treatment.

A sponsored clinical trial is currently being conducted in Italy (ClinicalTrials.gov identifier: NCT0244398; https://clinicaltrials.gov/ct2/show/NCT02443948) to establish an association between the change in cfDNA and disease state of GISTs. Another recent study using next-generation sequencing (NGS) successfully detected secondary *KIT* mutations in circulating cfDNA from the peripheral blood of GIST patients undergoing imatinib therapy [51].

## QClamp™ mutation assays using cfDNA

**Fig. 4:**
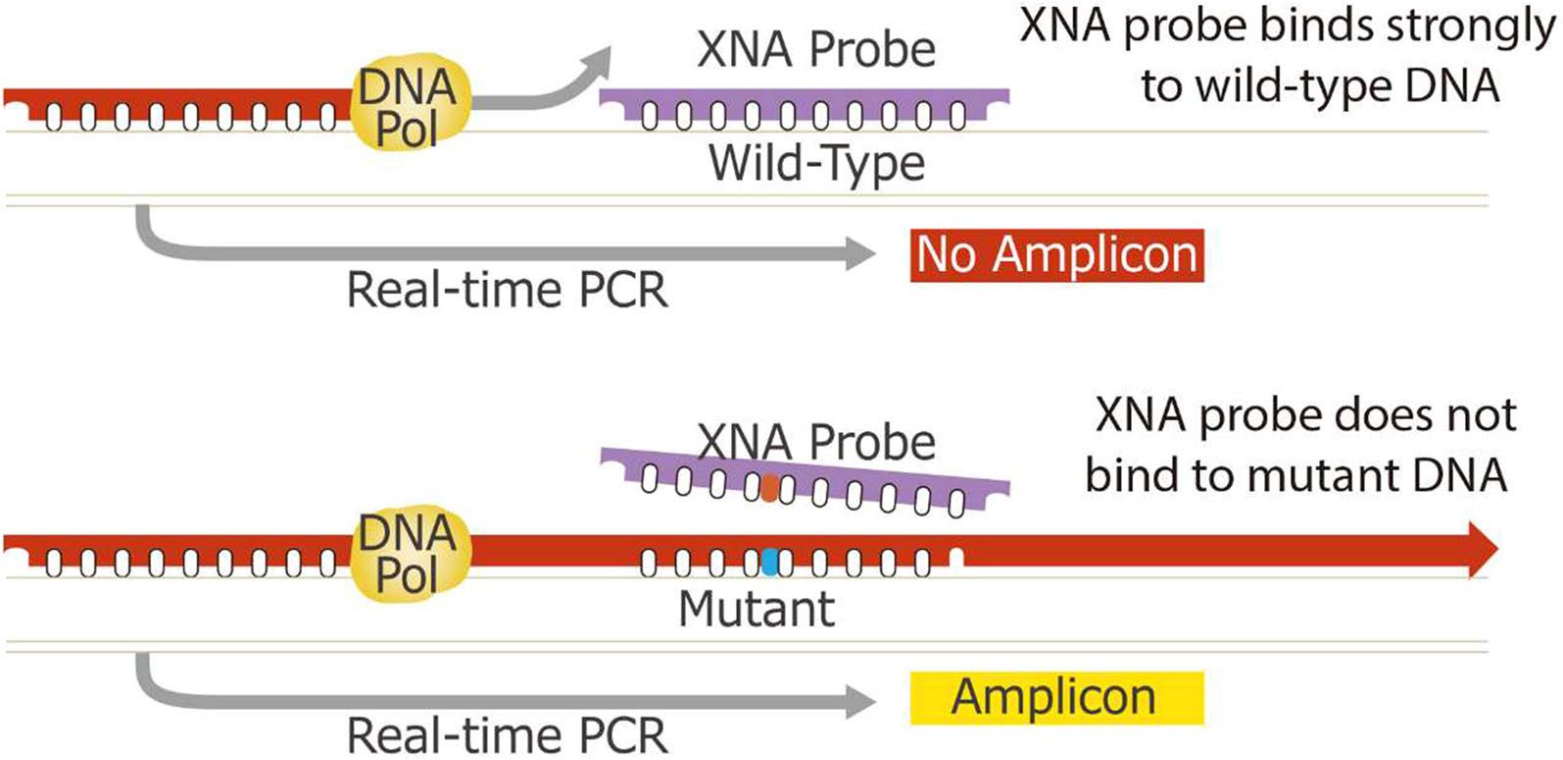
QClamp™ xenonucleic acid (XNA) probe hybridizes to wild-type DNA (WT-DNA) templates and prevents WT-DNA from being amplified, while melting off from the mutant DNA template so that only mutant DNA is amplified

For the high-sensitivity detection of low-frequency mutations in tumor-derived cfDNA, a technology has been developed that uses a polymerase chain reaction xenonucleic acid clamping approach (XNA-PCR; QClamp™). This novel technology has been developed for the detection of low-frequency mutations in tumor-derived DNA [52] and the early detection of mutations in cfDNA from cancer patients [53] and from stool-derived DNA in colorectal cancer patients [54]. This technology allows for the selective primer-mediated DNA polymerase-based amplification of only mutant template in tumor-derived DNA because the wild-type templates are blocked by the highly specific XNA probes used during PCR (Fig. 4).

**Fig. 5:**
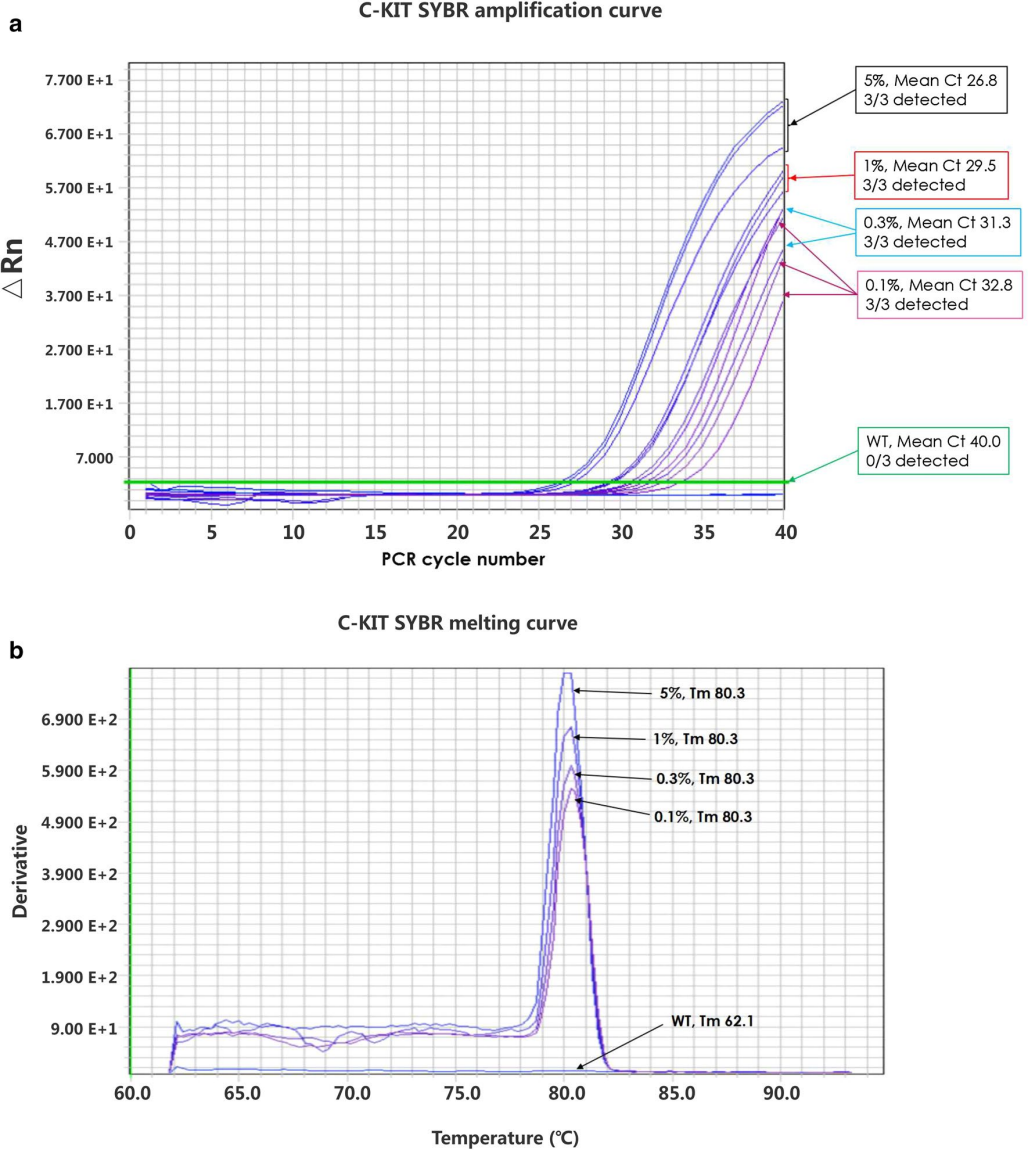
QClamp™ assay for *KIT* exon 17 D816V/Y/H mutation detection performed with template DNA containing D816V mutations at the allelic frequencies shown. **a** Real-time polymerase chain reaction (PCR) profiles *KIT* D816V amplification plots. **b** Melting curves for PCR amplicons clearly demonstrate detection sensitivity of >99.9% (i.e., 1 mutant allele in 1000 wild-type alleles)

Mutations in growth factor receptor pathways can guide a physician in choosing the appropriate precision therapy for a particular cancer. QClamp™ can detect mutations in tumor-derived cfDNA from plasma samples. The QClamp™ method is highly sensitive and can detect mutations at a level below 0.1% (1 mutant copy in 1000 wild-type copies). For example, *KIT* mutations in exon 17, particularly at codon 816, lead to activation of the kinase in GISTs. Figure 5 shows the real-time PCR profile for the high-sensitivity detection of this mutation at <0.1%.

The QClamp™ XNA-PCR technology has also been used to detect low-frequency mutations in breast cancer patient samples [55] and fine needle aspiration (FNA) biopsy samples [56], which further demonstrates the practical clinical use of the technology for detection of low-frequency mutant tumor alleles in tumor biopsy samples.

## Conclusions

The prognosis for GIST patients has changed enormously over the past few decades. In particular, imatinib has radically changed life expectancy of patients with GIST, a group of patients previously considered to be largely untreatable. For those with disease refractory to imatinib, as well as the majority who develop resistance to imatinib, other TKIs, such as sunitinib and regorafenib, are available. As more drug options become available, mutation testing by pathologists will become more common to select the best drugs for treatment depending on the characteristics of the tumor, whether the treatment is for primary tumors or not, and whether there are metastases that are present at diagnosis or that develop during treatment.

The high sensitivity of QClamp™ technology and its ability to be performed using widely available real-time PCR instrumentation platforms such as ABI 7500, Roche LC480, and Rotor-Gene Q make it highly attractive for clinical applications where a rapid “sample-to-answer” result is needed for precise medical applications. It is expected that the QClamp™ technology will find wide application in the minimally invasive detection of tumor-specific mutations to aid in the early diagnosis of GISTs and the development of targeted therapies.
